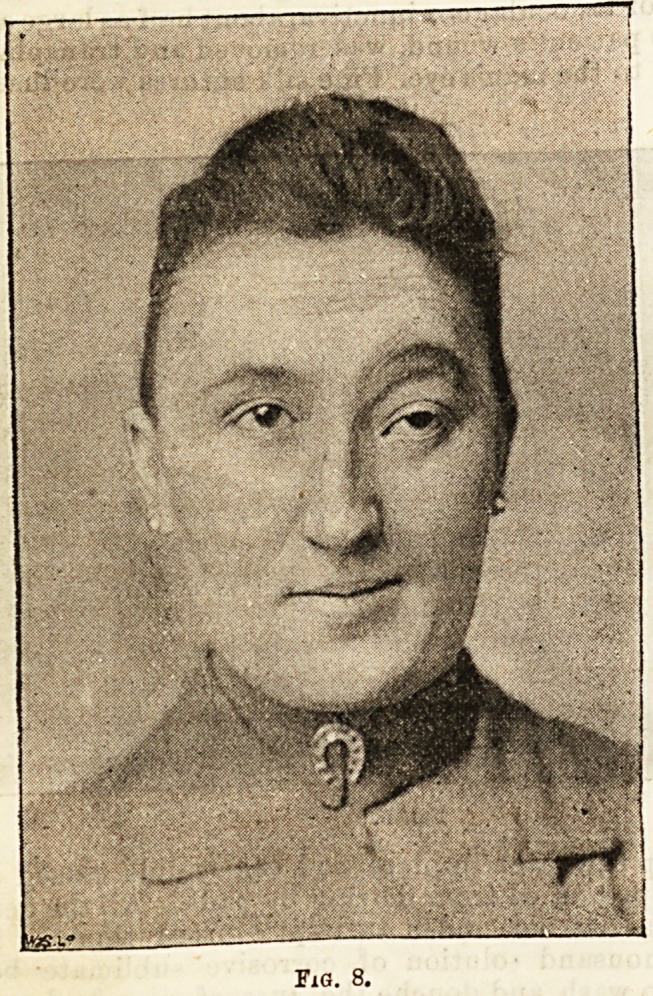# The Treatment of Cosmetic Defects—II

**Published:** 1893-06-24

**Authors:** 


### THE HOSPITAL CLINIC.

[ *The Editor will be glad to receive offers of co-operation and contributions from members of the profession. All letters should be addressed to* The Editor, The Lodge, Porchester Square, London, W.]

### BATH EYE INFIRMARY.

### The Treatment of Cosmetic Defects.—II.

It is not always easy to decide what the result of an operation for cosmetic purposes will be. It is a fine

Fig. 5.

art, and in order to be a successful surgeon in this direction artistic instincts are necessary. Science and art cannot be dissociated; the surgeon and the artist must combine their resources, the latter to say what is required, the former how it can be accomplished.

Take the case of a patient who has had an eye enucleated. Very few deformities can compare with that so produced. The prominent and sharply defined bony margins of the orbit, the concave retracted upper lid, the inturned lower lid disclosing the reddened mucous membrane, together make up a picture highly suggestive of the charnel house. In such a case the cosmetic effect of a well-fitting glass eye is magical: it is the difference between death and life. A glass eye has, however, advantages beyond the mere cosmetic ones. It keeps the lids in their proper position, thus preventing entropion with its consequent irritation from the inturned lashes, and it also acts as a barrier against the entry of dust and dirt of all descriptions. And this brings us to the next point of consideration—namely, that an operation is very seldom merely an advantage cosmetically. Usually the attempt to imitate or reproduce the appearance of nature, if successful, is rewarded by a reproduction to some extent of other natural conditions, for the connection between operations for cosmetic and those for optical and other effects is a very close one. A cosmetic operation may be defined as one, the object of which is to produce an imitation of the appearance of nature, and it follows that the more nearly we can reproduce a natural appearance the more likely are we to restore other normal conditions.

In considering the question of operation, it is very important to remember, and to point out to the patient, too, the possibility of failure, in which case a still further deformity may be produced. This applies especially to plastic operations, in which a portion of healthy skin or conjunctiva is removed or partially removed and patched into a faulty or disused new site. Should the patch necrose, it is quite likely that an increased disfigurement will result to the ever-visible damage of both patient and surgeon. A female patient will never forget or forgive such a contretemps, and, therefore, too much consideration cannot be given to the possibility of failure before operating in such cases. The pros and cons having been pointed out, the sensible patient will rely upon the surgeon to weigh them in the balance, so that it is usually impossible for him to divest himself of all responsibility in the matter.

Cosmetic operations are justified then not only on æsthetic grounds, but also because in many of them other associated advantages accrue. A successful result may mean to the patient the difference between following his accustomed trade or profession and beginning life again with a new one. The friends, too, who are daily brought into contact with the victim of an ocular deformity should have some consideration shown to them; it is not pleasant to dine side by side with a man with ectropion and epiphora, or opposite a woman with well-marked strabismus.

Some of the most troublesome cases to deal with from a cosmetic point of view are those of symblepharon. Do what we will some amount of deformity almost always remains, and even with prolonged supervision contraction frequently follows and mars the result. Fig. 5 represents a man whose eyes were injured by an explosion in a South Wales colliery. Both eyes were tied down by cicatricial bands connecting the cornea with the lower lids. In his case the bands were divided and dissected back, exposing a diamond-shaped raw surface. A rabbit having been chloroformed, a piece of its ocular conjunctiva, about half as large again as the patient's wound, was removed and transplanted direct to the man's eye. Fine silk sutures were inserted

Fig. 6.

to keep the patch in place and the eyelids closed over it and prevented from moving by pads. All the details were carried out under antiseptic precautions, a four per thousand solution of corrosive sublimate being used to wash and douche the eyes of man and rabbit both before and during the somewhat prolonged operation. Two days after the eyes were opened and the conjunctiva was found to be in good apposition. The sutures were removed and the lids closed again as before. The result is seen in Fig. 6, which was taken six months after the operation. He can use his eyes much more freely, and the remains of the objectionable red bands are hidden by the lower lids.

Fig. 7.

Fig. 7 shows the displacement of an eye downwards and outwards caused by an exostosis growing from the inner wall of the orbit. Details of this case have been

Fig. 8.

published in the twelfth volume of Trans. Ophth. Soc. The disfigurement was very great and threatened to interfere with the girl's occupation, that of a nurse-maid. Removal of the growth enabled the eye to sink back into its natural position in the orbit, but its function as a seeing organ was seriously and permanently impaired. Fig. 8 shows the same patient nine months after removal of the exostosis, and two years later there was no recurrence of the growth.

Although operations which are undertaken for cosmetic purposes hold a very secondary position when compared with those the object of which is to save life or to restore vision, yet they cannot be considered too trivial to deserve careful thought on the part of the surgeon. Failure may not mean death, but it is very likely to cause increased deformity. The utmost delicacy and finesse are requisite, therefore, in the details, and a foresight as to what the ultimate result is likely to be when healing is complete and contraction at an end.

Under any circumstances, one does not care to meet in one's daily walks persons with deformities of the eyes, and it would not mend matters if the surgeon thought he saw in those eyes a sinister look, which seemed to say, "You, sir, are the cause of my affliction."

## Figures and Tables

**Fig. 5. f1:**
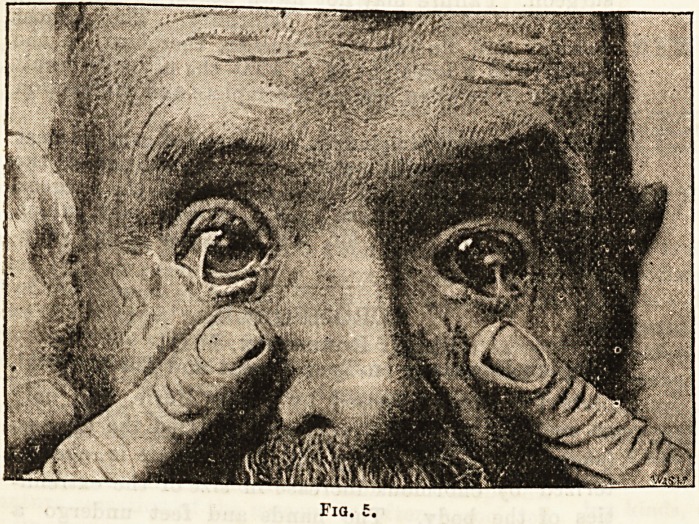


**Fig. 6. f2:**
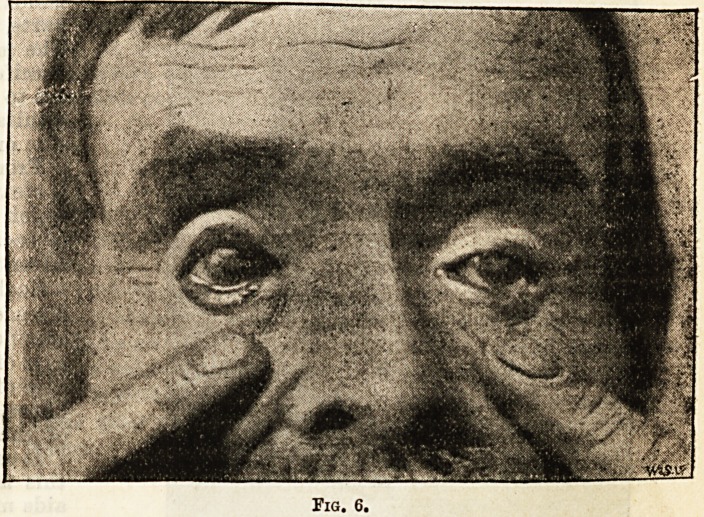


**Fig. 7 f3:**
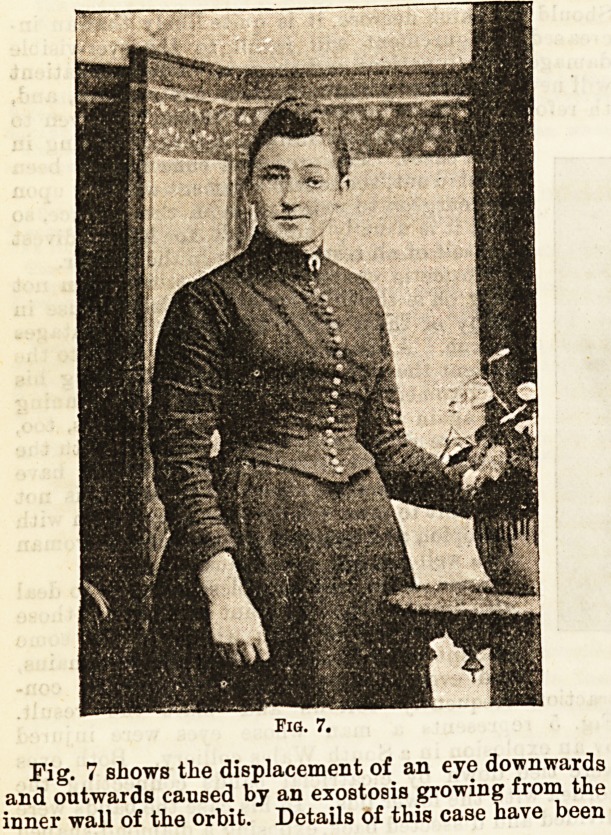


**Fig. 8. f4:**